# Standardizing the reporting of Mendelian randomization studies

**DOI:** 10.1186/s12916-023-02894-8

**Published:** 2023-05-18

**Authors:** Shiu Lun Au Yeung, Dipender Gill

**Affiliations:** 1 School of Public Health, LKS Faculty of Medicine, The University of Hong Kong, Hong Kong Special Administrative Region, China; 2 Department of Epidemiology and Biostatistics, School of Public Health, Imperial College London, London, UK

**Keywords:** Checklist, Mendelian randomization, Reporting, STROBE-MR

## Background

Mendelian randomization (MR), i.e., instrumental variable analysis using genetic instruments, is increasingly used in epidemiologic investigations to improve causal inference within an observational study design. This paradigm is more robust to environmental confounding and reverse causation than traditional epidemiological study designs and can be implemented using summary statistics from genome wide association studies (GWAS) [1]. For example, BMC Medicine has already published 11 MR studies between 1 January and 5 April 2023, in comparison to only 4 studies published in the year of 2018. In view of this trend, BMC Medicine has recently updated the submission guidelines where authors of MR studies are strongly encouraged to report their studies according to the STROBE-MR checklist (https://bmcmedicine.biomedcentral.com/submission-guidelines/preparing-your-manuscript/research-articles) [2]. It is hoped that their standardized reporting is of benefit to journal editors, reviewers, and readers for critically appraising the evidence and facilitating its interpretation.

## STROBE-MR checklist to improve reporting of MR studies

Reporting of MR studies has been variable, hence limiting potential for appraisal [3]. Consequently, the STROBE-MR checklist (https://www.strobe-mr.org/) has been developed to help authors ensure reporting of all the details essential for evaluating the quality and validity of an MR study [2]. The checklist includes 20 main items and 30 subitems covering different aspects, ranging from assessing underlying assumptions and characteristics of the underlying GWAS, to the reporting of corresponding findings [2]. Reporting based on STROBE-MR will hopefully help authors ensure that all relevant elements of the MR study are considered. This would also help avoid pitfalls (e.g., lack of allele harmonization) that can drastically impact the validity of the study [4].

However, more checks on the STROBE-MR checklist do not necessarily mean the MR study is of better quality. For example, authors may inaccurately report assumptions for sensitivity analyses or use wrong unit for exposures. Furthermore, a credible MR study requires careful design by the authors. Examples of additional considerations include those related to selection bias for diseases having a late age of onset (e.g., Alzheimer’s disease) [1]; possibility of substantial biasing pleiotropy related to genetic variants for particular phenotypes (e.g., observed drug use); the choice of GWAS (e.g., only relying on data curated in particular databases which may not be most up-to-date and hence may limit statistical power); and relevance of instruments in the outcome GWAS (e.g., the use of smoking intensity instruments among smokers only). Conversely, not all elements of the checklist may be applicable to every type of MR study. For example, where a single unweighted genetic variant is used as an instrument, details of the statistical methods used to generate the MR estimate are less applicable, as these are only variant-trait associations.

## Other forms of Mendelian randomization studies and emerging methodologies

The STROBE-MR checklist primarily focuses on conventional MR studies that assess the impact of an exposure on a disease outcome. However, there are emerging forms of MR studies (e.g., drug-target MR studies) that require reporting of additional information, which has only been touched upon briefly in STROBE-MR [2]. Specifically, drug target MR studies investigate the effects of drug target perturbation by leveraging variants in the putative gene regions as instruments [5]. As a result, this form of MR requires reporting of additional information, such as clear description of the gene region, the trait used to identify genetic instruments, corresponding MR methods, and whether the analysis was corroborated with colocalization analyses [6]. Similarly, MR studies now also increasingly consider circulating proteins as exposures. This form of MR study has different selection criteria for instruments. Specifically, *cis*-protein quantitative trait loci (*cis*-pQTL), defined as the variants in the protein encoding gene region, are often preferred as they are generally less prone to having pleiotropic effects as compared to trans-pQTL variants, which are selected from throughout the genome rather than the corresponding protein’s gene region. Such MR study often also includes colocalization to assess whether a distinct variant at a locus explains the genetic associations with two traits. Conventional sensitivity analyses such as weighted median and MR-Egger are likely inappropriate given these *cis*-variants are highly correlated and hence are susceptible to the same degree of bias from pleiotropic associations. Authors using these various MR designs should therefore consult respective guidelines or recommendations for proper reporting regarding the items in STROBE-MR checklist [5, 7]. Furthermore, MR methods have been evolved rapidly and authors should be mindful of the new methodologies which may outperform previous methods. One recent example is the use of doubly ranked stratification method instead of residual based method when assessing non-linear effects with MR [8].

## Conclusions

In conclusion, adherence to STROBE-MR checklist will likely improve reporting of MR studies for better evaluation and interpretation by those involved in the peer review process, as well as the end users. Authors of MR studies should be aware of any additional information needs to be reported based on the respective designs for proper assessment. Via triangulation of evidence, well-conducted MR studies can complement findings from traditional epidemiological studies and randomized controlled trials, collectively contributing to a more solid evidence base for public health policies and prioritization of clinical trial study for communicable and non-communicable diseases [9, 10].

## Data Availability

Not applicable.
